# Hygiene and Health: Who Do Mothers in Vanuatu Communicate with about Health?

**DOI:** 10.3390/ijerph15030443

**Published:** 2018-03-03

**Authors:** Karen File, Thomas Valente, Mary-Louise McLaws

**Affiliations:** 1 School of Public Health and Community Medicine, UNSW Sydney, Sydney 2033, Australia; karenafile@gmail.com; 2 Department of Preventive Medicine, Institute for Prevention Research, University of Southern California, Los Angeles, CA 90007, USA; tvalente@usc.edu

**Keywords:** health-seeking, handwashing, behaviour, social network, urban, rural, traditional, water

## Abstract

Health information-seeking behaviour of mothers with children five years of age and younger in Vanuatu was examined using the structural properties of social networks. Data were collected from a rural village from two islands and an urban settlement in the capital, Port Vila, by face-to-face interviews using a structured questionnaire. Sociometric data on the structure of the network, the characteristics of key informants, and associations with outside sources of health information were analysed as interpersonal predictors of health promotion and behavior change. Rural mothers preferred the health advice of biomedical practitioners for diarrheal disease over traditional custom practitioners. Interpersonal connections were restricted in the urban mother network indicating that mothers were merely acquaintances or do not seek health advice from each other. Our findings suggest that biomedical practitioners are the best option for diffusing health and hygiene information for rural and urban mothers. Traditional healers and paraprofessionals could be strategically used to complete the missing links in network connectedness to optimally spread new information. The novel use of cross-sectional social network data can create a baseline evaluation to purposefully frame a health intervention. Our study provided a unique explanation of how network analysis offers insight into how key players can be identified and the circumstances in which they are likely to be able to influence hygiene practices of their peers.

## 1. Introduction

Diarrhoea accounts for 8% of all deaths across the globe in children under five years of age [1]. The Vanuatu Multiple Indicator Cluster Survey [2] found diarrhoea in 14% of children under five years of age and little urban (12.8%) and rural (14.1%) difference was observed [3] (p. 19). The Ministry of Health (MoH) survey found no association between diarrhoea prevalence, education and the economic status of mothers ([3], p. 19). Handwashing is a potentially effective means of reducing the burden of disease in children but there has been little improvement in domestic handwashing in the past 30 years [4,5,6,7]. Health promotion researchers appreciate that changing handwashing behaviour is complex and difficult [8,9,10]. Behavioural and social scientists report that the critical psycho-social influences on personal handwashing behaviour include disgust with dirt, parental upbringing, social norms and discomfort with washing [11,12,13,14,15,16,17,18,19]. According to the social learning and diffusion theory, the key driver to change behaviour at the individual, interpersonal and community level relies on social norms [18,20]. Individual (intrapersonal) behaviour is often associated with social norms including the behaviour of friends, family members and associates who engage in the target behaviour [21]. Intrapersonal factors include the individual attributes that impact behaviours such as “knowledge, attitude, beliefs and personality traits” ([12], p. 3). Interpersonal factors involve relationships or communication with others (family, friends and peers) providing social identify, social status and social norms. Social cognitive models attempt to capitalise on both interpersonal and intrapersonal determinants of behaviour [12].

The dimension for changing social norms is an in-depth understanding of how individuals are situated within the social network of their community [16,18,22,23]. The social network approach focuses on the community network and their relationships with people occupying key positions, also known as key players, in the network that may afford opportunities or constraints for behavioural interventions [24]. The use of opinion leaders has successfully promoted evidence-based practise [25]. Their network would enable them to influence the opinions, attitudes, beliefs, motivations and behaviours of others [26]. Opinion leaders improve dissemination of the desired behaviour and accelerate behaviour within the network. In the case of handwashing practice, the social network approach could be successfully leveraged by first identifying opinion leaders and key individuals to influence adoption.

The use of social networks to diffuse target information has been successful in changing community health practice [27], and family planning [22]. In the past, communication about handwashing has focused on when and why to undertake the behaviour in low and lower-middle income settings [18]. Yet, the most efficient method for moving knowledge about this behaviour through a community’s health advice network is missing: knowledge transfer via diffusers. Handwashing interventions in households have had limited success [14,28], and would benefit from an understanding of knowledge transfer. Social network analysis identifies opinion leaders to diffuse knowledge and behavioural influence efficiently throughout advice networks [25,29]. Using the approach of network positions, we examined health advice-seeking to determine how best to diffuse handwashing through the community.

We have examined the health information-seeking behaviour of mothers in a rural and urban community in Vanuatu where routine handwashing for the purpose of disease prevention is not routinely practiced. Quantitative social network analysis was used to understand how mothers with children five years of age and younger seek general and health advice, and interpersonal predictors of health promotion and behaviour change. We sought to capture community-level advice-seeking interactions of mothers that are comprised of deep social relations between family and members of the community. A combined health-seeking social network was used to examine: the nature of connections between the mothers; the overall shape and structure of the network and the characteristics of key informants; and clustering of the network and associations with outside sources of health information.

## 2. Materials and Methods

### 2.1. Study Design

A network analysis was performed on cross-sectional data. Participants were mothers with children five years of age and younger who lived in an urban informal settlement in Port Vila and in the rural coastal community of “Rural 1” situated in the north of Efate Island in Vanuatu. “Urban 1” and “Rural 1” communities were purposively chosen for their central location and comparability to other community in Vanuatu. Ethics approval (HC10394) was given by the UNSW Sydney (University of New South Wales), Human Research Ethics Committee and the Vanuatu Ministry of Health Ethics Committee. Community consent was given by community leaders and participants. Participants signed a written consent form approved by the UNSW Human Research Ethics Committee and the Vanuatu Ministry of Health Ethics Committee.

### 2.2. The Setting

The Republic of Vanuatu is an archipelago in the South Pacific Ocean with a population of about 234,000 [30], of whom the majority live a subsistence rural lifestyle.

The rural community of “Rural 1” is a small traditional rural coastal village on Efate island in Vanuatu with approximately 300 inhabitants. “Rural 1” is located on a major road, 15 min by car from the nearest health centre in Panganisu and one hour from the 86 bed Vila Central Hospital in the Port Vila, Vanuatu. One volunteer female village healthcare worker manages the “Rural 1” village aid post and administers basic healthcare and health advice. “Rural 1” has 98% Ni-Vanuatu population with a socio-political landscape that is hierarchically organised on sex and age [30]. The village is typically Christian with the largest denomination being Presbyterian. The introduction of Christianity by missionaries did not remove traditional ‘kastom’ belief and practice. A synchronistic system of kastom and Christianity permeates every aspect of daily life [31].

Port Vila has high population growth with a burgeoning of informal settlements in urban and peri-urban areas. “Urban 1”, located in Port Vila, is one of the oldest informal urban settlements in the capital, established in the late 1970s. It comprises three separate communities typically defined by home island group: Tongoa, Paama and Futuna demark geographic settlement boundaries. “Urban 1” has approximately 606 residents comprised of households from two islands [32] with an average household size of approximately 5.5 people (range 1–2, maximum 9–10) [32]. Most houses are constructed from any available material including corrugated iron, wood and recycled construction material [32]. The primary mode of transportation is by foot, private mini-bus or private transport.

### 2.3. Participant Selection and Questionnaire

Our investigation of advice networks of mothers with children five years of age and younger followed Rogers et al.’s [33] approach to communication networks of Korean women from 25 village in the 1970s. Mothers were chosen because they are said to be at a “life-stage” that is receptive to change proponents and customarily responsible for children’s physical care (toileting and feeding) and protection [34], are believed to be the best prospect for future prevention program [14], and teach their children handwashing behaviour as a self-protection practice [35].

The mothers were informed of the purpose of the project and details of methods. They assisted in drawing a community map detailing the homes of all mothers with children five years of age and younger. Data collection occurred during August 2012 in “Rural 1” and June 2011 in “Urban 1” communities. A trained research assistant who had family in “Rural 1” village, held a high school certificate and was fluent in the Bislama language invited every mother with children five years of age and younger who resided in the target communities to participate in the research.

Structured social network questionnaires were administered to all target eligible consented mothers living in “Rural 1” village and “Urban 1” via census. Social network data were collected during a face-to-face interview in either the mothers’ homes or yards adjacent to their homes.

Because two people might be connected in a general advice-seeking network but disconnected in a health advice-seeking network [36], participating mothers were asked to nominate up to four people from their community who they go to for general and health advice. This study did not explore relationships between nominations outside the community; rather, a closed community network was examined with mothers nominating advice relationships within the village regardless of the parental status of those nominated. Mothers were asked about hearing information from outside the community but were not asked to nominate relationships outside the community. A questionnaire from a study using social network analysis to understand health-seeking associated with family planning [22] was modified with permission to accommodate place names and appropriate demographic questions. The combined advice network includes mothers’ community advice-seeking for general and health information. Participants named up to four people they seek advice from, in accordance with Stoebenau et al. [22]. The questionnaire was piloted in the Club Hippique area located in peri-urban Port Vila. The modified questionnaire was divided into two sections (available on request):

1. Demographic characteristics of mothers: occupation, education, religion, marital status
2. Social network items:
  a. General advice inside the community: Q15 *Have you talked to anyone in “Rural 1” village/”Urban 1” to get advice or discuss problems in the past six months?*
  b. Health advice inside the community: Q23 *Have you talked to anyone in “Rural 1” village/”Urban 1” to get health advice in the past six months?*
  c. Health information and advice outside the community: Q34 *What other ways have you heard about personal health, other than from people “Rural 1” village/”Urban 1”?*; Q35 *Of the people who live or work outside “Rural 1/”Urban 1”, whom have you talked with about personal health in the past six months?*

### 2.4. Network Analysis

**Figure 1:**
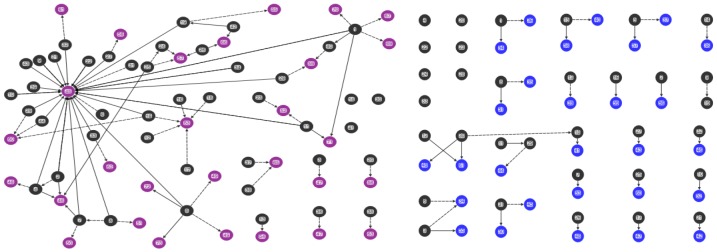
Combined mothers’ health advice-seeking network (Q15, Q23). “Rural 1” left hand side and “Urban 1” right side. Black nodes indicate mothers with children five years of age and younger, purple nodes indicate nominations, black ties denote health advice nominations and black dashed ties denote general advice nominations.

**Figure 2:**
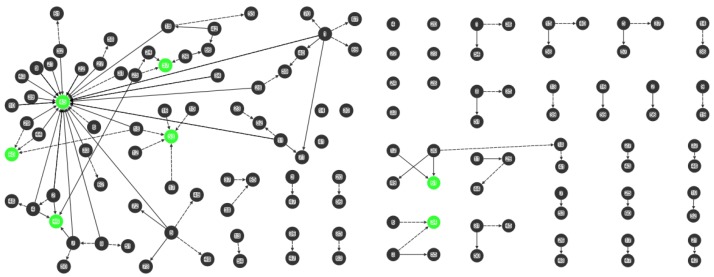
Centrality in the combined mothers’ health advice-seeking network (Q15, Q23). “Rural 1” left hand side and “Urban 1” right side. Green nodes indicate individuals with high in-degree scores (greater than three), black ties denote health advice nominations and black dashed ties denote general advice nominations.

**Figure 3:**
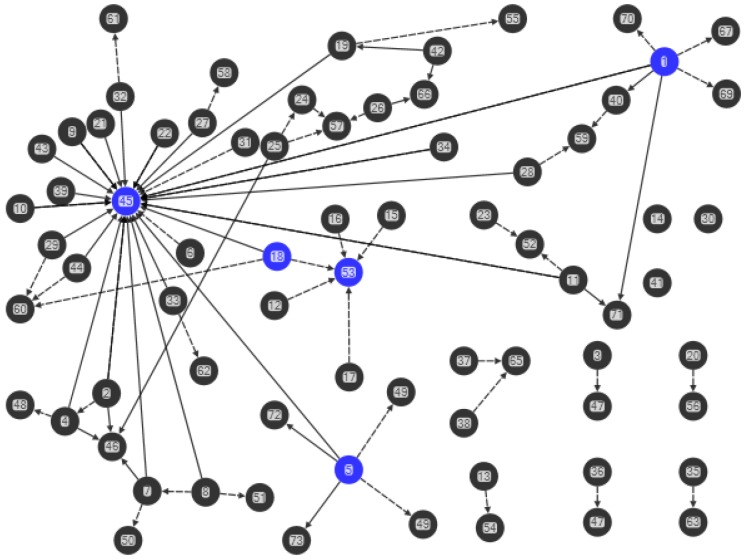
Brokerage in the combined mothers’ health advice-seeking network (Q15, Q23). “Rural 1” left hand side and “Urban 1” right side. Blue nodes indicate individuals with high betweenness centrality, black ties denote health advice nominations and black dashed ties denote general advice nominations.

**Figure 4:**
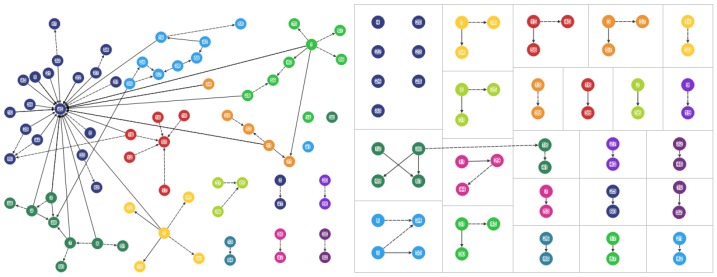
Clusters in the combined mothers’ health advice-seeking network (Q15, Q23). “Rural 1” left hand side and “Urban 1” right side. Coloured nodes denote the clusters, black ties denote health advice nominations and black dashed ties denote general advice nominations.

Data were analysed using NodeXL software (Social Media Research Foundation, Belmont, CA, USA) [37] and examined by demographics and for sociograms [33,38,39]. Sociograms generated from NodeXL [37] were used to map the mothers’ advice networks, and health knowledge and behavior (Figure 1, Figure 2, Figure 3 and Figure 4). Nodes represent the actors in the communication network, the lines represent the relational ties of seeking advice, and the arrows show the direction of information-seeking. In Figure 1, Figure 2, Figure 3 and Figure 4, every line (relationship) has an arrowhead (direction of information-seeking). Advice networks are asymmetric: person *x* seeks advice from person *y* but person *y* does not seek advice from person *x* [36]. Confidentiality and discretion were maintained by coding individuals using numbers.

Four sociograms capturing interpersonal advice relationships, and knowledge and behavior were developed:

1. “Combined mothers’ health advice-seeking network” (Q15, Q23): both general and health advice-seeking (Figure 1)
2. “Centrality in the combined mothers’ health advice-seeking network” (Q15, Q23): individuals with high in-degree measures (Figure 2)
3. “Brokerage in the combined mothers’ health advice-seeking network” (Q15, Q23): individuals with high betweenness measures (Figure 3)
4. “Clusters in the combined mothers’ health advice-seeking network” (Q15, Q23): pockets of interconnected individuals (Figure 4)

The following measures were calculated to describe the attributes of people and networks:

**Density** measures the connectedness of mothers to others in their community. Network density is measured by the number of ties present divided by the number of possible ties [40].

**Geodesic distance** measures the extent of connection in the network and is the shortest pathway between two individuals [41].

**Reciprocity** measures relationship strength and is the percentage of all ties in the network. Reciprocity of relationship is evident when people acknowledge the relationship by naming each other [36].

**Centrality** measures the most connected people by measuring those who interact with the most other people. Connected people are important potential diffusers in networks and hold highly influential positions in a network. Two measures of centrality were calculated for each person. Degree centrality indicates status and was calculated as the number of ties as a proportion of the number of people in the network [42].

**In-degree** and **out-degree** is measured by the number of advice relationships *to* and *from* an individual, respectively [43]. In-degree is used to identify opinion leaders in a network and measure social integration [36].

**Betweenness** measures the extent to which an individual lies on the shortest path (geodesic distance) to others who are not connected [43]. Betweenness centrality identifies brokers or bridges. Brokerage is the extent to which an individual plays a connecting role between two distinct groups or clusters. Brokers can add substantive value to networks by generating new ideas and making advice and knowledge more accessible to others. Brokers act as gatekeepers controlling the information passing into and out of their cluster or network. They may bottleneck information but generally they are positively associated with diffusing useful information [44].

A **cluster** measures the degree of structure within the network. A cluster is a “subgroup of a network in which the local density of ties is higher than across the whole network” [45]. A network with high clustering indicates dense pockets of interconnectivity; conversely, low clustering has few groupings of connectivity [36]. The average personal density for all the nodes in a network is the clustering coefficient [46].

**Tie strength** measures the emotional intensity, level of reciprocity or frequency of interaction associated with a tie (link) between two people [45]. At the individual level, strong ties are important for behavioral adoption, whereas at the network level, weak ties are important for transmitting information [47]. The strength of weak ties lies in the effective spread of new information from outside of one’s close relationships (who all tend to know the same things) [47]. Weak ties may actively diffuse new information because they connect people who as individuals are not normally connected [47,48]. Weak ties are measured in two different ways: structurally and relationally. In this study, structural measures of weak ties are derived from sociometric data and weak ties are individuals with links spanning the structural holes or distantly connected groups in the network. **Isolates** are people with no connections inside the network who are disconnected from the network [36].

## 3. Results

### 3.1. Demographic Characteristics of the Study Sample

In “Rural 1” village, 98% (44/45) of mothers who received a questionnaire were successfully interviewed. All 44 participants had attended primary school, 59% (26/44) had attended high school, and all were stay-at-home mothers who worked in their family garden. In the “Urban 1” community, 97% (33/34) of mothers who received a questionnaire were successfully interviewed. Levels of education and socio-economic status were low. Mothers had either primary or secondary education; only one (individual-4) had tertiary level education. Forty-six per cent of mothers attained a secondary level of education. Mothers were generally (73%, 25/34) “stay-at-home mums”.

### 3.2. Sociograms

Health advice-seeking for the combined networks of mothers showed ties that were only of those mothers within either the rural or urban communities. Figure 1, Figure 2, Figure 3 and Figure 4 correspond to the “combined health advice-seeking network”, and “centrality”, “brokerage” and “clusters” within the network, respectively. Mothers with no ties sought advice from outside the community or did not seek advice at all.

### 3.3. Combined Advice-Seeking (Q15, Q23)

Most (93%, 41/44) “Rural 1” mothers identified up to four peers inside their community for their general and health advice-seeking. Only three mothers had no ties within the network. The combined advice network (Q15, Q23) of 73 mothers had 69 unique interpersonal relationships (either general advice, health advice or both) and 352 advice relationships from inside the mothers’ network (Figure 1). The 352 advice relationships denote the possible number of nominations (edges) and was calculated from 44 mothers who responded to four general nominations and four mothers who responded to four health nominations. The network is a common wheel or star structure and characteristically has a large variance in individual degree scores (in-degree range 1–25; out-degree range 0–7) suggesting that one person controls most of the activity [49]. This network has a low density, 1.4%, and reciprocity was not apparent. The average geodesic distance, that is, the shortest path length, between individuals was three. Mothers rarely (except individual-5) sought advice from other mothers. The network illustrates a traditional seeking pattern of the rural healthcare worker (individual-45) who had the highest nominations for health advice (in-degree-25), while a church leader (individual-53) and a chief (individual-60) were most frequently nominated for general advice (Figure 2).

In “Urban 1”, all mothers identified four or fewer peers as their sources of advice. The combined advice network (Q15, Q23) consisted of 62 people who had 31 unique interpersonal relationships for advice and 132 advice relationships (Figure 1). This network was small and highly disconnected. It had an extremely low density of 9%. The average geodesic, shortest, distance between individuals was one. The network was characterised by dyadic relationships consisting of two individuals and reciprocity was not apparent across the network.

Information-seeking is localised. A large number of mothers seek advice from inside the “Rural 1” community from people who hold a variety of community roles: friends (individuals-35, -36, -38, -41, -48, -49, -62), family (individuals-34, -39, -43, -44, -46, -49, -50, -51, -52, -53, -54, -55, -56, -57, -58, -59), church relations (individual-47), a healthcare worker (individuals-42, -60) and a chief (individual-45). Mothers seek health advice most commonly (81%, 36/44) and seek it from female family members who do not have children five years of age and younger.

### 3.4. Central Individuals

In the “Rural 1” network (Q15, Q23), each person had an average of one person as their source of advice (in-degree 1.630, range 0–25) (Figure 2). For health advice, 25 of the 44 participants nominated healthcare worker-1 (individual-45) (Figure 2). The former village health worker-2 (individual-46) had the second highest in-degree score of four for health advice.

In “Rural 1”, five people (individuals-45, -46, -53, -57, -60) were most sought after for advice by three or more other people while the remainder were nominated once or not at all. Mothers seek advice from people with a variety of community roles: the current and former village health workers (individuals-45, -46), family (individual-57), the church (individual-53) and a chief (individual-60) (Figure 2).

In “Urban 1”, central individuals have the largest in-degree measurement, the number of people seeking advice from them. In the combined network (Q15, Q23), each person had an average of one person as a source of advice (in-degree 1.065, range 0–2) (Figure 2). Only two (individuals-34, -61) were most sought after for advice by two other people (in-degree 2) while the remainder were nominated once or not at all.

### 3.5. Clusters

In “Rural 1”, the average clustering coefficient for the combined advice-seeking network is 2.7% (range 0–50%). The combined advice network had 14 clusters of relationship (Figure 4). The one dominant star cluster present was the current village health worker (individual-45) while a former village health worker had a smaller weak cluster. People who occupy central positions (individuals-45, -46, -53, -57, -60) link individuals within clusters, while bridging people (individuals-1, -5, -18, 45, -53) link clusters within the network. All but one cluster (with individual-45) had a small number of connections.

In “Urban 1”, the average clustering coefficient for the combined advice-seeking network was too low to calculate. This network was very low in clustering with poor interconnectivity.

In “Urban 1” the combined advice network had 22 clusters of relationship and the largest cluster was comprised of four people. Consultations in this cluster included friends (individuals-49, -61) and another participant mother with a child five years of age and younger (Figure 3). The remaining clusters were comprised of 14 dyadic and six triadic relationships.

### 3.6. Tie strength, Brokers and Isolates

In “Rural 1”, the network was characterised by a significant number of weak ties that included individual brokers spanning isolated clusters inside the network. Several respondent mothers held dual roles (individuals-45, -53) as opinion leaders and brokering information across the network. Some respondent mothers were isolates with only a link outside the network.

The village health worker-1 (individual-45) in “Rural 1” had the highest betweenness centrality followed by three mothers (individuals-1, -5, -18) and a church leader (individual-53). Betweenness centrality for network was not calculated because the connections in this network were about the nomination of advice experts and not directly meaningful to this network. The majority of mothers (89%, 39/44) had connections with outside sources for either general or health advice (Q35). Thirty-three of these individuals (75%) seek advice from the nurse practitioners at the health centre 15 min away. Other sources of advice were families living outside “Rural 1” and a community radio program (Q20). Three of the 39 mothers (individuals-1, -5, -18) who had outside advice sources held brokerage positions for the potential to bring in new health information to the network.

Across the 44 “Rural 1” participants, three (individuals-14, -30, -41) were isolates who only seek advice from outside the community network (Figure 3) by consulting the nurse practitioners in Location.

The “Urban 1” mothers’ network was characterised by weak ties. Many of the possible relationship links were absent. The highly decentralised network for combined advice-seeking network meant that a calculation of the average betweenness centrality score was not possible (by the software NodeXL). There was an absence of intermediary actors to facilitate transactions between other individuals within the network.

Many of the “Urban 1” mothers seek only general advice or only health advice or no advice by three isolates (Figure 3). Two isolated mothers (individuals-4, -22) seek advice from family outside the “Urban 1” network, two seek advice from the health workers at Vila Central Hospital (individuals-23, -28) and the remaining three (individuals-20, -24, -33) did not seek advice of any kind.

Of the 33 “Urban 1” mothers, 11 (33%) nominated an outside individual for advice from family outside the area and health workers at the hospital. Health information from outside the community was delivered via health awareness or health workshops (23%, 3/11), doctors (18%, 2/11), nurses (20%, 2/11), and variety of minor sources (36%, 4/11) included posters pamphlets, radio, friends and family.

## 4. Discussion

Networks play an essential role in the information transmission process. Utilising the network structure and function in health communication strategies has been shown to be effective in the adoption of knowledge and health behavior [39].

### 4.1. Relationships

The “Rural 1” sociograms suggest an element of trust implied in mothers seeking advice from within their community and comprised of deep social connections (with kastom and church leaders and others). Social trust is indicative of social capital or, conversely, social constraint [36]. Trust, usually significant in this type of network, is a prerequisite condition for advice-seeking and interpersonal influence and is solidified with advice-seeking interaction [50]. These connections manifest as social capital in the form of information about who has power and resources in the community [36]. Mobilising mother’s advice-seeking relationships in the “Rural 1” group could ameliorate the health and hygiene situation of children aged between newborn and five years old.

The “Urban 1” sociograms indicate that interpersonal connections are restricted. The data indicates at best that the set of people in this network are merely acquaintances or do not seek advice of any kind. A number of factors may account for this network being small and highly disconnected. In “Urban 1”, residents are faced with the difficult meeting point of traditional and modern living [32,51]; that is, tradition and the web of traditional relationships are being eroded [51]. As a highly restricted urban network, the “Urban 1” mothers’ network highlights the trend away from traditional life and the problem of meeting the obligations of Western living, urbanisation and the cash economy. With changes in ‘contemporary social life’, young mothers are vulnerable to social isolation [51]. The “Urban 1” mothers’ advice network highlights the crisis of urbanisation for young mothers in urban informal settlements in Port Vila.

### 4.2. Density of Relationships

Community group mothers seek advice about their problems and children’s health on a needs basis [52]. Consequently, the level of diffusion of knowledge and behavior in the “Rural 1” advice network is low because sparse relationships are less efficient at diffusing information across the network than a dense network. Yet, the more network relationships there are, the greater the difficulty in maintaining these relationships and efficiency [53]. Relatively low density and short path lengths make the “Rural 1” community advice network optimally efficient [54]. Fewer relationships between individuals may increase the adoption of new health behaviors because of the strengthened influence of the health experts in the networks on the target health issues [36]. Too much density can constrain the formation of links or bridges to external information and people [36].

### 4.3. Central Individuals

In “Rural 1”, half (22/44, 56%) of all the mothers sought health advice from the village health worker. The village health worker was given the highest nominations and the one central dominant star cluster. Therefore, this health worker could be considered an expert source of health information within the community and the preferred source of advice before outside medical advice from the health centre with the potential for overload and reduced productivity over time. A study by Fujimoto et al. [55] on community leadership and prevention strategies found that utilising people in the informal discussion network increased behavior adoption. Using the opinion leaders could enhance communication within the “Rural 1” community, starting with the formal sources and then the less formal sources starting with the religious leader and the chief. In “Rural 1”, people providing general advice could be used to provide mothers with more choices for obtaining health information and increase the flow of health information [36].

The “Urban 1” sociograms indicate that five people (family and mothers with children five years of age and younger) were the only central individuals with any likelihood for diffusing advice on health knowledge and behavior. Centrally positioned important figures were not identified, which highlights an absence of hierarchy and lack of knowledge across the network [36].

### 4.4. Clusters, Brokers and Ties

One dominant star cluster characterised the “Rural 1” network, centred on health advice-seeking, and a small weak cluster of relationship was centred on a former village health worker. Five brokers, including the village health worker, three mothers and a church leader, were identified to diffuse information, and create cohesion and build social capital [21]. “Rural 1” is coherent, rich in weak ties, so that new ideas spread rapidly, linking family, belief systems and place [48].

A large proportion 33/44 (75%) of individuals not linked to the “Rural 1” network sought health information from the health centre in the village located 15 min away. These weak ties create cohesion and spread information [36,47,48]. They could support the controlled transfer of health and handwashing knowledge between the inside and outside of the network, thereby increasing cooperation and efficiency by liaising with people from both sides [44].

The “Urban 1” mothers’ network has low personal network densities and poor interconnectivity with weak clustering. Normally such low rates of clustering are indicative of a network that is easier to penetrate than a network with cohesive subgroups. However, with little interconnectivity, which is needed to spread knowledge and behavior, diffusion in the “Urban 1” mothers’ network is problematic. A lack of sufficient clustering and an absence of brokerage roles within the network suggest that social capital is poor [21]. This network is disconnected with no evidence of integrated knowledge, skills or resources, indicating that it is functioning at a minimum. The present configuration and poor social capital in the “Urban 1” mothers’ network would greatly restrict any benefits of brokerage.

### 4.5. Practical Implications and Recommendations for Health Communication and Behavior Change

#### 4.5.1. Opinion Leadership

The “Rural 1” mothers’ advice-seeking network is a centralised structure that is suited to opinion leadership strategies [33]. Valente suggested, “Local leaders [in community networks] provide advice that is more sensitive to local conditions and culture” ([29], p. 52)*.* The current healthcare worker-1 influence may be derived from trust found through family relationships and to mothers, limiting relationships to a small group and people (in general) who are similar to themselves with respect to culture, values and concerns [36,39,56]. In this study, for minor illness in small children advice was sought from the village healthcare worker. In the case of severe illness, the health centre or hospital was the preferred option, providing higher levels of treatment and care [52].

#### 4.5.2. Change Agents

The “Rural 1” network could be altered to include traditional healers to first diffuse information and resources and then contribute to targeted behavior change [29]. Maden et al. [57], in their study on alternative medicine use at Vila Central Hospital, asked patients and staff about their use of kastom (traditional custom) medicine. Twenty-one medical patients (42%) had used kastom medicine for their current problem (12% while in hospital). Eighteen surgical patients (36%) had used kastom medicine for their current problem (6% while in hospital). Fourteen of the staff (28%) interviewed had used kastom medicine within the last year. The use of kastom medicine is common across education levels with a trend for those with higher education to use less kastom medicine. Traditional healers (klevas) acquire or inherit their knowledge to diagnose and treat social and health problems using prayer, massage and prophylaxis [58]. Traditional healers are important individuals in the village system; they work within the norms and framework of kastom [57]. In the context of handwashing, traditional healers could be added to the network to bridge disconnected individuals or groups, their homogeneity facilitating information diffusion, decision-making and behavior change.

The constraint governing the “Urban 1” mothers’ network highlights the need to develop linkages from outside the network. Deploying outside change agents or lay health advisors to link (bridge) the health system (change agency) and the individual (patient or client) could bring new information into the network and address the limited health communication across the network [21,29,44]. In rural Vanuatu, village health workers recruited as change agents (paraprofessional aides) increase staffing in health care across rural Vanuatu. As a paraprofessional (recruit completing a particular aspect of a professional task), the cost per client contact is much lower than a professional recruit, a critical factor for a low-income country [21]. They are also socially closer to the lower-status members of the user system that they serve. Individuals from the broader community could be selectively recruited and trained as “urban” health workers replicating the rural health worker model to provide adequate access to health services in Vanuatu.

### 4.6. Social Capital and New Knowledge

The “Urban 1” mothers’ network is low in social capital and trust, and consequentially has few existing resources that could be mobilised by the network. Szreter reports “where urban neighbourhoods and rural communities (and particular sub-populations) are demonstrably low in social capital, residents report higher levels of stress and isolation, children’s welfare decreases, and there is a reduced capacity to respond to environmental health risks and to receive effective public health service interventions” ([59], p. 651). Kawachi [31] suggests, “closer ties with neighbours can have a net negative effect on the health of residents, especially in deprived communities”. “Within disadvantaged communities [such as Seaside], stronger bonding ties may involve higher expectations to assist neighbours in need, and hence higher levels of financial and mental strain” ([60], p. 991). The key to promoting health in “Urban 1” could be for mothers to be able to access resources outside the confines of their social setting. Expanding the quality and quantity of vertical relationships that link healthcare promoters and mothers could address the restricted flow of new information, and increase social capital. Linking social capital via change agents (such as paraprofessional aides) could provide access to new health and handwashing information and resources from outside the network and within the community.

### 4.7. Limitations

Although these findings in a specific target group in a rural and urban community may not be generalisable to other rural communities, they provide support for the diffusion approach to spread health information where social norms for the targeted behavior are missing. Other potential biases that might have resulted in measurement error include terminology used, selection of mothers and caregivers rather than the full community, or those who did not participate in the study due to lack of interest or relevance, or being too busy to participate. Even though this study provided explanations of the meanings of some terms like “advice” and “health” in the questionnaire, there remains the possibility that participants misunderstood the terms, and diversity in understanding of the meaning of these terms cannot be excluded as a threat to internal validity of observed patterns. It may be that respondents were concerned about confidentiality and did not report truthfully those they sought advice from.

## 5. Conclusions

Health promoters would benefit from using “the network as a delivery vehicle but also us[ing] network information to learn from the community to better serve community needs” ([36], p. 49). Network data simplifies the complexity of interpersonal advice relationships at the individual and whole network level through quantitative analysis. Contrary to recent research in Vanuatu on therapeutic preference for local traditional healers [57,58], this study found that “Rural 1” mothers prefer biomedical practitioners over traditional kastom practitioners. Biomedical practitioners are possibly the best option for diffusing maternal child health and information. Traditional healers and paraprofessionals as brokers are also critical, optimally spanning the holes in network connectedness, without the need to maintain the status quo, and so strategic for diffusing new information. If network interventions are to be culturally sensitive, local traditional healers must also be included in evidence-based approaches in the development of the intervention [61].

The idea of using social networks to diffuse a health behavior is not novel [36] but this example of how cross-sectional social network data can be used to create a baseline evaluation and purposefully frame a health intervention is. Social network methods have not been published in a traditional rural community setting in Vanuatu (located in the western Pacific Ocean). Unexpectedly, this study found that urban and rural mothers rarely seek advice from their peers, opening other opportunities for diffusing health behaviors. This study shows how network analysis offers the opportunity to determine whether and how key players can be identified and the circumstances in which they are likely to influence the health practice of their peers.
